# Novel Bovine Serum Albumin-Decorated–Nanostructured Lipid Carriers Able to Modulate Apoptosis and Cell-Cycle Response in Ovarian, Breast, and Colon Tumoral Cells

**DOI:** 10.3390/pharmaceutics15041125

**Published:** 2023-04-02

**Authors:** Robert Tincu, Mirela Mihaila, Marinela Bostan, Florina Teodorescu, Daniela Istrati, Nicoleta Badea, Ioana Lacatusu

**Affiliations:** 1 Faculty of Chemical Engineering and Biotechnologies, University POLITEHNICA of Bucharest, Polizu No. 1, 011061 Bucharest, Romania; 2 “C.D. Nenitzescu” Institute of Organic and Supramolecular Chemistry of the Romanian Academy, 202B Splaiul Independentei, 060023 Bucharest, Romania; 3 Stefan S. Nicolau Institute of Virology, Mihai Bravu Street No. 285, 030304 Bucharest, Romania; 4 Faculty of Pharmacy, Titu Maiorescu University, 040314 Bucharest, Romania; 5 Department of Immunology, Victor Babes National Institute of Pathology, 050096 Bucharest, Romania

**Keywords:** hybrid albumin-lipid nanocarriers, piperine, fluorescence properties, tumor cell apoptosis

## Abstract

A novel nanoscale approach was developed for the improved cellular internalization of hybrid bovine serum albumin–lipid nanocarriers loaded with piperine (NLC-Pip–BSA) in different tumor cells. The effect of the BSA-targeted–NLC-Pip and untargeted-NLC-Pip on the viability, proliferation, and levels of cell-cycle damage and apoptosis in the colon (LoVo), ovarian (SKOV3) and breast (MCF7) adenocarcinoma cell lines was comparatively discussed. NLCs were characterized concerning particle size, morphology, zeta potential, phytochemical encapsulation efficiency, ATR-FTIR, and fluorescence spectroscopy. The results showed that NLC-Pip–BSA showed a mean size below 140 nm, a zeta potential of −60 mV, and an entrapment efficiency of 81.94% for NLC-Pip and 80.45% for NLC-Pip–BSA. Fluorescence spectroscopy confirmed the coating of the NLC with the albumin. By MTS and RTCA assays, NLC-Pip–BSA showed a more pronounced response against the LoVo colon cell line and MCF-7 breast tumor cell lines than against the ovarian SKOV-3 cell line. Flow cytometry assay demonstrated that the targeted NLC-Pip had more cytotoxicity and improved apoptosis than the untargeted ones in MCF-7 tumor cells (*p* < 0.05). NLC-Pip caused a significant increase in MCF-7 breast tumor cell apoptosis of ~8X, while NLC-Pip–BSA has shown an 11-fold increase in apoptosis.

## 1. Introduction

Biopolymers have been assigned major importance in medical and pharmaceutical applications. Currently, intensive work is being done to identify hybrid delivery systems that will manifest more advanced therapeutic effects. The physical, chemical, and biological characteristics (well established) allow certain categories of biopolymers to be successfully used in different biomedical sectors. The concept of combining biopolymers with lipid-based delivery systems (prepared with physiologically compatible lipids as skeleton materials) represents the latest research. Such examples include solid nanoparticles [1], lipid nanocarriers [2], liposomes, niosomes, etc., [3]. The hybrid lipid carriers that are coated or functionalized with biopolymers, e.g., hyaluronic acid, pectin, chitosan, and albumin have numerous advantages, such as physical stability, biodegradability, low toxicity, and high drug-loading capacity [4]. For example, Shehata et al. demonstrated that pectin-coated lipid nanocarriers loaded with piperine exhibited high entrapment efficiency, physical stability, controlled release, and enhanced cytotoxicity against hepatocellular carcinoma compared to uncoated NLCs [5].

The importance of using a biocompatible carrier material has been well recognized for the potential clinical translation of nanomedicine. In the physiological environment, nanoparticles (NPs) can interact with numerous components, which may alter the performance of delivery systems, including morphology, diameter, and stability. For example, physiological proteins could be adsorbed onto the surface of NPs during circulation and form a coating called a “protein corona”. The non-specific NP interactions with the various blood components lead to the prevention of NP adsorption and produce an activation of the immune response [6]. For this reason, the initial coating of NPs with hydrophilic polymers will lead to a reduced protein-adsorption effect (from the physiological environment) and thus protect their recognition by the reticuloendothelial system. Therefore, a “stealth effect” approach is covering the NPs with a hydrophilic polymer that inhibits their non-specific interactions with blood components, avoiding the activation of an immune response and ensuring an extension of the circulation time in the body [6]. In this context, albumin has been intensively explored to construct albumin-based NPs and other albumin nanocarrier systems with improved biomedical functions.

**Figure 1:**
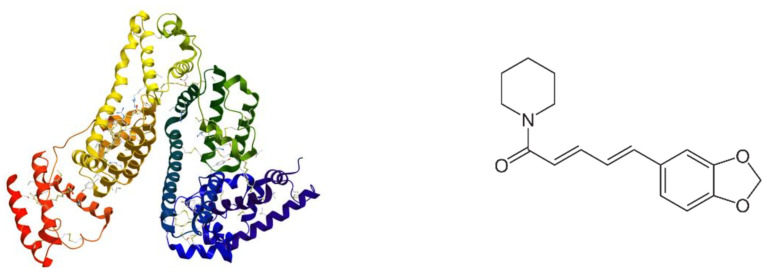
Structure of BSA and Pip.

Bovine serum albumin (BSA) is the most abundant water-soluble plasma protein, whose main physiological role is to maintain blood pH and osmotic pressure [7]. Structurally, it consists of 583 amino acid residues; it has three α-helical regions (I, II, III), each containing two separate helical subdomains (Figure 1): A (4 α-helices) and B (6 α-helices) [8]. As a blood-circulating protein and a transporter for various exogenous compounds within the vasculature, albumin shows a relatively long circulation half-life and favorable biodegradable and biocompatible characteristics [9]. It has a half-life of about 19 days in the blood, which improves the pharmacokinetic profile of drug-loaded albumin-based-NPs [10]. Albumin NPs have gained considerable attention owing to their high capability to load several drugs as well as their tolerability when administered in vivo [11]. Albumin’s in vivo safety has been demonstrated through its applications in clinical nanomedicine [12,13]. There are many advantages of albumin NPs to facilitate their clinical applications and the development of clinically approved albumin-related formulations, Abraxane (Paclitaxel albumin nanoparticles approved for metastatic breast cancer, non-small cell lung cancer, and pancreas adenocarcinoma) is the most excellent example [12]. Encouraged by the success of Abraxane, the development of various albumin-based NPs for drug delivery and bioimaging could guarantee the biocompatibility and the clinical approval of new nanostructured carriers. In the field of drug delivery, albumin could be used as a coating biopolymer, template, scaffold, and nanocarrier [13,14,15,16,17,18,19,20]. Covalent conjugation and non-covalent assembly are involved in the preparation strategies of the various kinds of albumin-based NPs. For instance, in Abraxane, the preference is for a non-covalent assembly because it exerts no effect on the chemical entity of albumin [12]. Owing to the amino and carboxylic groups, albumin also participates in non-covalent assembly, i.e., the electrostatic adsorption of positively or negatively charged molecules, obtaining a variety of functionalities such as prolonged half-life in circulation, improved stability, prolonged drug release, or targeted release [14]. Albumin–lipid NPs consisting of medium-chain triglycerides and a lactobionic acid–human serum albumin conjugate were developed by Dayani et al., 2022 as a targeted drug-delivery system of Sorafenib for the treatment of hepatocellular carcinoma [15]. Albumin-based NPs synergistically facilitated the mRNA and siRNA delivery with improved anti-tumor action [16]. In another related study, albumin-coated lipid NPs were prepared and evaluated for their gene downregulation effect, through the delivery of siRNA to breast cancer cells [17]. According to the latest research, Wang et al., 2022 developed lipid-coated albumin–paclitaxel NPs loaded with sorcin and siRNA for suppressing cancer chemoresistance via restoring intracellular calcium ion homeostasis [18]. In these and other research studies, nanosized albumin is covered by lipids, i.e., dioleoylphosphatidylethanolamine/DOPE [18], or albumin is conjugated with polysaccharide to form novel colloidal drug carriers, i.e., albumin–hyaluronic acid colloidal nanocarriers [19,20] or lipid–albumin nanosystems by liposome type [17,21]. To the best of our knowledge, there are no studies in the scientific literature that address the coating of solid delivery systems such as nanostructured lipid carriers (NLC) with bovine serum albumin. In one related research study, in silico albumin corona around solid lipid nanoparticles (SLN) loaded with curcumin and capsaicin were produced [22]. Another study focused on the impact of particle size and pH on the formation of BSA corona–solid lipid nanoparticles (SLNs) [23].

Piperine (Pip) is an herbal alkaloid obtained from the plants, such as black pepper and long pepper, belonging to the *Piperaceae* family, and is called the “king of spices”. This bioactive alkaloid exhibits a wide range of beneficial physiological and pharmacological activities. It has excellent therapeutic efficacy against a variety of ailments, including neuronal disorders and anticancer action e.g., hepatocellular carcinoma or breast cancer [24]. However, its low aqueous solubility (40 mg/L at 18 °C), extensive first-pass metabolism, and toxicity at high concentrations limit its therapeutic applications and clinical use [25]. Some nanocarriers have been employed to improve the dissolution and oral bioavailability of Pip and to enhance their anticancer efficacy: chitosan based-nanoparticles for brain cancer [26], PEG-PLGA nanoparticles for breast cancer [27], emulsions for colon cancer [28]. A study by Shehata et al., looked at Pip-loaded-NLC and pectin-NLC-loaded with Pip targeting liver cancer. The obtained results showed an enhancement of the in vivo antitumor effect of Pip, the suppression of liver enzymes, and the oxidative stress environment in the liver [5].

Considering all the previously mentioned, it is meaningful to assemble lipid nanocarriers and albumin in a rational way (by non-covalent assembly, i.e., electrostatic interactions and hydrogen bonds) to build optimized nanoplatforms for augmenting the effects of phytochemical Pip-guided cancer therapy. Therefore, the goal of this study was to evaluate the effect of the BSA targeted-NLC-Pip and untargeted-NLC-Pip on the viability, proliferation, and levels of cell cycle damage and apoptosis in the colon (LoVo), breast (MCF7), and ovarian (SKOV-3) adenocarcinoma cell lines. The tumor cells suppressed by the conventional NLC-Pip and hybrid NLC-Pip–BSA were analyzed and compared to two well-known cytostatic drugs, Cisplatin and Doxorubicin. We did not find reports in which the cytotoxic action of the potential antitumor phytochemical, Pip, versus that of conventional chemotherapeutic drugs were compared.

## 2. Materials and Methods

### 2.1. Materials

The surfactants, Phosphatidylcholine (PC), Tween 20 (TW 20), Dioctyl sulfosuccinate sodium salt (AOT), lyophilized Bovine serum albumin (BSA) and the bioactive principle, Piperine (Pip) were purchased from Sigma Aldrich Chemie GmbH (Munich, Germany). Glycerol monostearate (GMS) and Cocoa butter (CB) were acquired from Cognis GmbH (Monheim am Rhein, Germany) and SoLaris (Bucharest, Romania), respectively. The vegetable oil used for building the lipid core, thistle oil (TO) was bought from Textron Plimon S.L.U. (Barcelona, Spain). The cytostatic drugs, Cisplatin (Cis-diammineplatinum(II) dichloride, Cys-Pt) and Doxorubicin (DOX) were purchased from Sigma Aldrich (St. Louis, MO, USA).

### 2.2. Cell Cultures Conditions and Treatments

The human cell line: LoVo (human colon adenocarcinoma), MCF-7 (human breast adenocarcinoma), and SKOV-3 (human ovarian adenocarcinoma) were bought from American Type Culture Collection (ATCC) and processed as described in Supplementary Material Section S1.1. The reference used was a normal cell line, human umbilical vein endothelial cells (HUVEC).

### 2.3. Preparation of Hybrid BSA-Coated Nanostructured Lipid Carriers

The optimized free and loaded NLCs were prepared by a modified high-pressure homogenization (HPH) described in previous studies by the authors [29,30]. Briefly, the aqueous phase containing the mixture of surfactants AOT, TW 20, and PC, heated at 72 °C was added to the melted lipid phase (also at 72 °C) consisting of GMS, CB, TO, and the bioactive principle, Pip. Total lipid phase concentration was kept constant at 10%. The composition of NLCs formulations is presented in Table 1A. The BSA-coated NLCs were obtained by mixing 20 mL of NLC-I/II/III with 20 mL of BSA aqueous solution of different concentrations, according to Table 1B, under high shear homogenization for one minute at 9000 rpm and ~40 °C. The solid hybrid–NLC formulations were obtained after the lyophilization of aqueous dispersions of NLCs, without the use of a cryoprotective agent (0.05 mbar, −55 °C, 54 h), using a Martin Christ Alpha 1-2 LD Freeze Drying System (Martin Christ, Osterode am Harz, Germany).

### 2.4. Characterization Methods

The mean particle size and polydispersity index were assessed using dynamic light scattering (DLS, Zetasizer Nano ZS, Malvern Instruments Ltd., Worcestershire, UK). The electrical characteristics of the samples were determined using electrophoretic light scattering. Transmission electron microscopy (TEM, Hitachi High-Tech Corporation, Tokyo, Japan) was employed to observe the morphological characteristics of the NLC-Pip–BSA samples. Spectral characterization was achieved by Fourier-transform infrared spectroscopy (FTIR, Bruker Vertex 70, Etilingen, Germany) and fluorescence spectroscopy (FP-650 Spectrofluorometer Jasco, Tokyo, Japan). The entrapment efficiency was determined by UV-Vis spectroscopy (UV-Vis Spectrophotometer V670 Jasco, Tokyo, Japan) [31,32]. In vitro activity of the prepared NLCs was studied using real-time cells assay (RTCA, ACEA Biosciences, San Diego, CA, USA), MTS assay (CellTiter 96 Aqueous One Solution Cell Proliferation Assay (Promega, Madison, WI, USA), and flow cytometry(FACS Canto II cytometers, Becton Dickinson, Immunocytometry System, Mountain View, CA, USA) [33,34,35,36]. For detailed characterization procedures see Supplementary Materials Section, S1.2.1. to S1.2.10.

### 2.5. Statistical Analysis

The study in question involved performing measurements in triplicate and analyzed the data using GraphPad Prism 7 software (GraphPad Software Inc., La Jolla, CA, USA). The differences between treatment and control groups, or between different treatments, were evaluated using one-way analysis of variance (ANOVA). Statistical significance was determined at a *p*-value of less than 0.05.

## 3. Results and Discussion

### 3.1. Optimization of Surfactants and Albumin in Obtaining Novel Biopolymer–Lipid Nanocarriers and Fluorescence Performance

**Figure 2:**
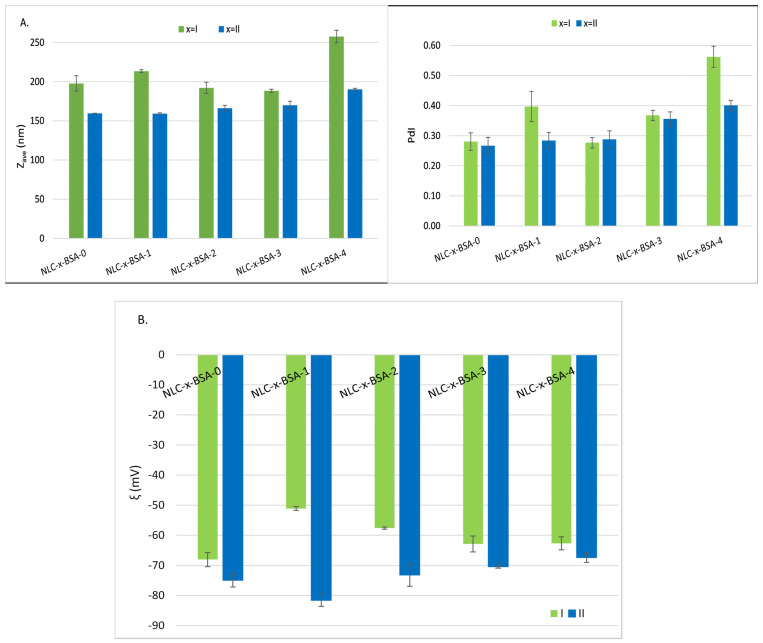
(**A**) Mean particle diameters and polydispersity index values obtained by DLS analysis for BSA−coated lipid nanocarriers; and (**B**) zeta potential values of BSA−coated lipid nanocarriers.

The preparation of lipid nanocarriers initially included the use of two categories of a surfactant blend i.e., NLC-I-BSA (consisting of Tw 20 and PC) and NLC-II-BSA (consisting of Tw 20, PC, and AOT) with the aim of creating an adaptable surface coating for the formation of weak bonds with the BSA biopolymer. Both AOT and PC could interact electrostatically as well as hydrophobically with BSA to form protein-decorated nanocarriers in the aqueous solution, in which the well-defined surfactant micelles are organized along the randomly distributed polypeptide chain of the BSA. The samples were processed at a pH of 4.5 at which BSA presents more positive charges favoring the electrostatic interactions with the anionic charges coming from anionic AOT and zwitterionic PC. The size and zeta potential of hybrid–lipid nanocarriers were tuned by using different weight ratios between surfactants and the BSA aqueous solution (Figure 2A,B). It was found that a surfactant fraction of Tw 20:PC: AOT = 1:0.7:0.3 in the protein-mixed surfactant complexes resulted in the formation of hybrid–lipid nanocarriers with average diameters of around 150 nm. An insignificant difference in mean diameter was detected with an increasing amount of BSA (Figure 2A). The same order of size magnitude was obtained by Wang et al. in a systematic insight into the impact of particle size and pH on BSA corona formation of solid lipid nanoparticles, i.e., with average diameters ranging between 134 and 449 nm [23].

The provision of adequate physical stability, which avoids coalescence, flocculation, or separation, involves the creation of a superficial layer that imprints values of zeta potentials > +25 or < −25 mV [32]. All hybrid biopolymer–lipid nanocarriers have appropriate physical stability, the zeta potential values were in the range of −52 mV to −80 mV (Figure 2B). According to the zeta potential and Z_ave_ results, the NLC-II-BSA stabilized by Tw 20-PC-AOT trilogy offers better dimensional and stability characteristics than NLC-I-BSA (prepared with non-ionic Tw 20 and zwitterionic PC). The zeta potential value of NLC-II-BSA (Figure 2B) was decreased compared to that of NLC-I-BSA, which is believed to be caused by better adsorption of surface charge BSA on the high electric surface charge of NLC, AOT being a contributing factor. This result argues the choice for the next experiments of the NLC-II-BSA formulations. As previously shown, BSA can establish mainly physical interactions like hydrogen bonds and van der Waals forces between the hydroxyl surfactant groups and with neighboring groups of amino acids residues of BSA [37].

**Figure 3:**
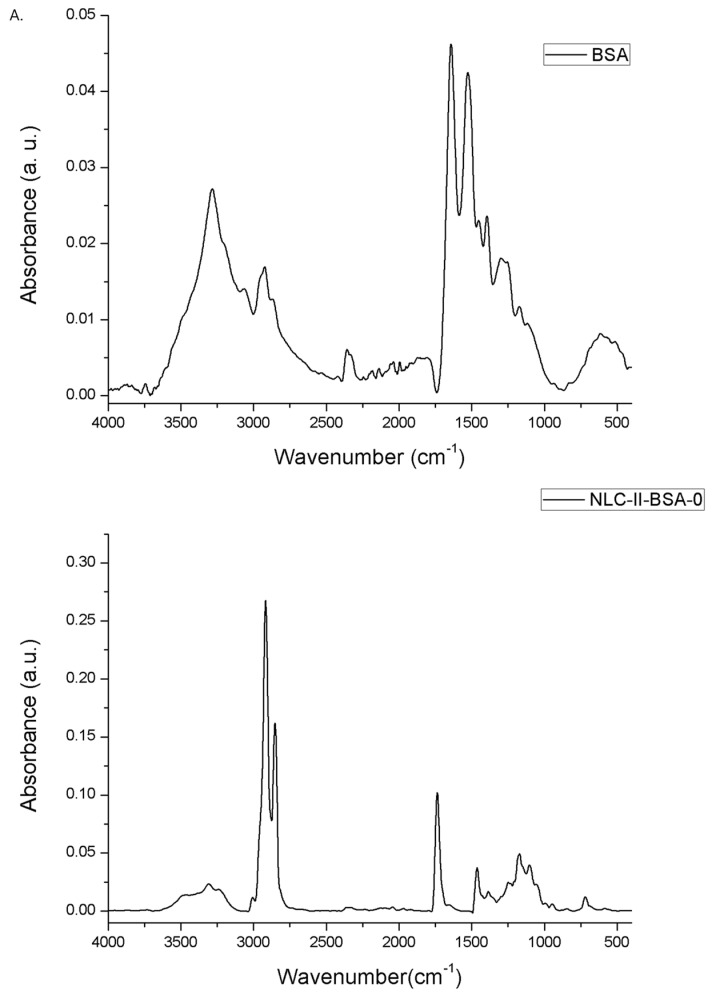

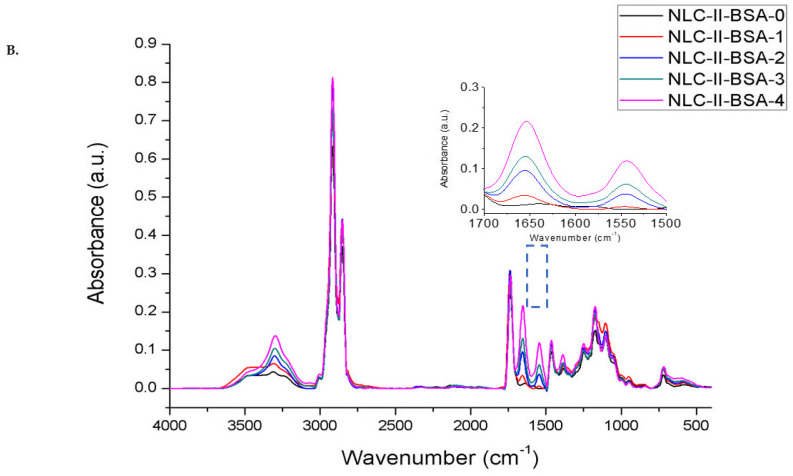
(**A**) ATR−FTIR analysis of albumin/BSA and NLC without BSA; and (**B**) ATR−FTIR analysis of albumin-coated lipid nanocarriers.

A comparative analysis of the ATR-FTIR spectra corresponding to NLC without BSA (NLC-II-BSA-0; Figure 3A) and the spectra of NLC-II-BSA-1/2/3/4 (Figure 3B) was conducted. Generally, all spectra exhibited similar characteristic bands corresponding to BSA and surfactants, as well as the increase in their intensity depending on the biopolymer concentration used to coat the NLC, for example, the C-H vibration (~3050–2830 cm^−1^) and C=O stretching (~1730 cm^−1^ for esters bond from AOT), and the amide I (1653–1656 cm^−1^) and II (1543–1546 cm^−1^) bands of BSA [38,39]. On the other side, some remarkable differences were found in the NLC with BSA (NLC-II-BSA-1/2/3/4) and the NLC without BSA (NLC-II-BSA-0). The intensity of vibration modes from ~3300 cm^−1^ and ~3450 cm^−1^ (angular deformation of O–H and N–H) was consistent with the appearance of the hydrogen bonds between the BSA and the spatially close functional groups of surfactants. Also, the strong O-H stretching (around 3300 cm^−1^) was observed to be intensified in NLC-Pip–BSA as compared to NLC-Pip, confirming that the hydrophilicity of NLC was greatly enhanced by the BSA coating. In addition, in the NLC-BSA spectra, the specific values of the amide I band were slightly shifted as compared to pristine BSA (1643 cm^−1^). These movements can be attributed to the changes in the protein structure (e.g., secondary structure change—increase in α-helix content), respectively, to the appearance of weak hydrogen bonds created between BSA and the functional groups of the surfactants used in the preparation of NLC. The changes in the protein structure are compatible with findings in the literature [23].

Fluorescence spectroscopy confirmed the coating of the lipid nanocarriers with the BSA biopolymer. BSA had a strong fluorescence emission band at about 330 nm, owing to its three intrinsic fluorophores, by means of the presence of aromatic amino acid-type residues such as *Trp* (emission wavelength at 348 nm), *Tyr* (303 nm) and *Phe* (emission 282 nm). According to findings in the literature, the main contributing factor to the intrinsic fluorescence of BSA is *Trp*, because *Tyr* is almost totally quenched if the functional groups are ionized or located near an amino or a carboxyl group [40].

**Figure 4:**
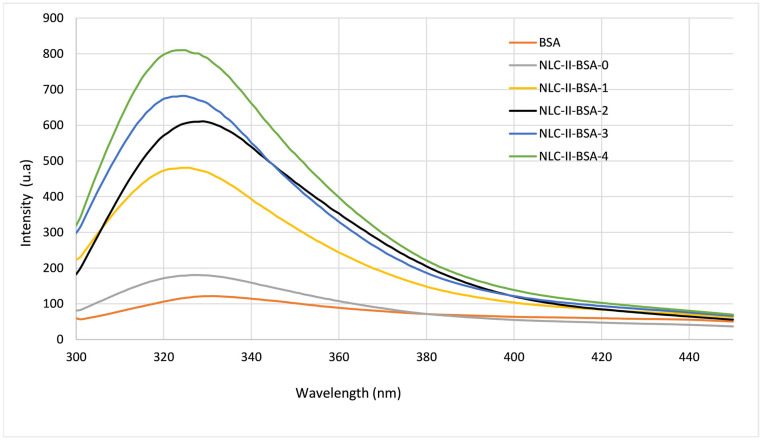
Fluorescence emission spectra of NLC-II-BSA-1/2/3/4 compared to NLC-free (NLC-II-BSA-0) and BSA.

NLC-II-BSA samples exhibited a significantly stronger emission profile than that of native/pure BSA (Figure 4). By associating BSA with NLC-type distribution systems, an enhancement of the fluorescence phenomenon can be observed as the concentration of protein used to coat the NLC was higher. For instance, for a weight ratio of 2.4:1 between BSA and ionic surfactant blend (AOT and PC) up to an 8-fold increase was detected in the maximum emission peak of NLC-II-BSA-4 as compared to BSA. This amplification can be attributed primarily to a size effect, the nanometric size of NLC covered with BSA (characterized by a large specific surface area) gives rise to the creation of more specific interaction centers, which in turn confer a more intense fluorescent emission. Besides the nano effect, the predominant factor responsible for fluorescence enhancement is the amount of BSA used to cover the NLC (Figure 4) associated with the type of molecular binding interactions which can be established between BSA and surfactants. Non-covalent hydrophobic interactions of BSA and Tween 80 have been suggested by Nishihira et al. when studying the BSA corona of curcumin–solid lipid nanoparticles [22].

### 3.2. Lipid Nanocarriers Coated with Bovine Serum Albumin and Loaded with Piperine

**Figure 5:**
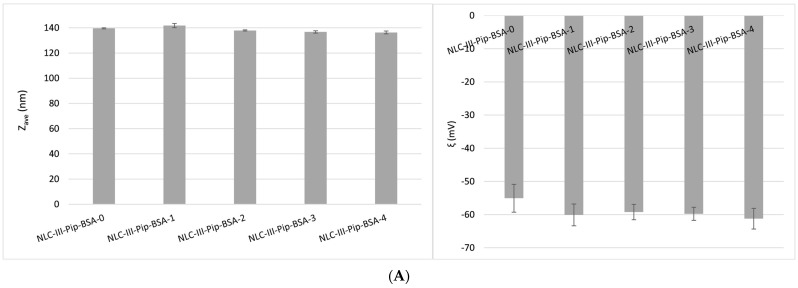

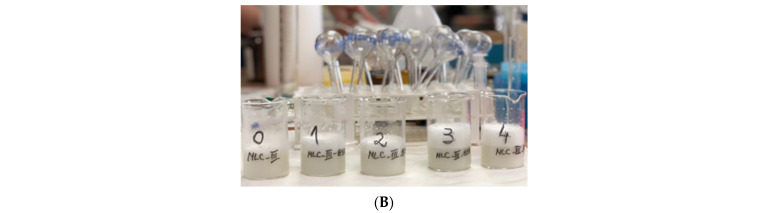
(**A**) Size and stability characteristics of BSA−coated lipid nanocarriers loaded with Pip; and (**B**) different experimental NLC−Pip−BSA formulations.

**Size and morphological aspects.** According to the DLS analysis, the mean particle size did not vary between formulations NLC-III-Pip-BSA-1/2/3/4 (Figure 5), prepared with different concentrations of BSA used to coat the nanocarriers. All the prepared hybrid–NLC presented a mean size below 140 nm, considered suitable for nanocarriers intended for drug delivery. In addition, the physical stability was maintained at a high level for all the BSA-coated–NLC-Pip, the registered zeta potential values being around −60 mV. Results were compatible with results obtained from the Fonseca et al. study for resveratrol-loaded BSA nanoparticles with zeta potential values ranging between −37 and −52 mV [41].

**Figure 6:**
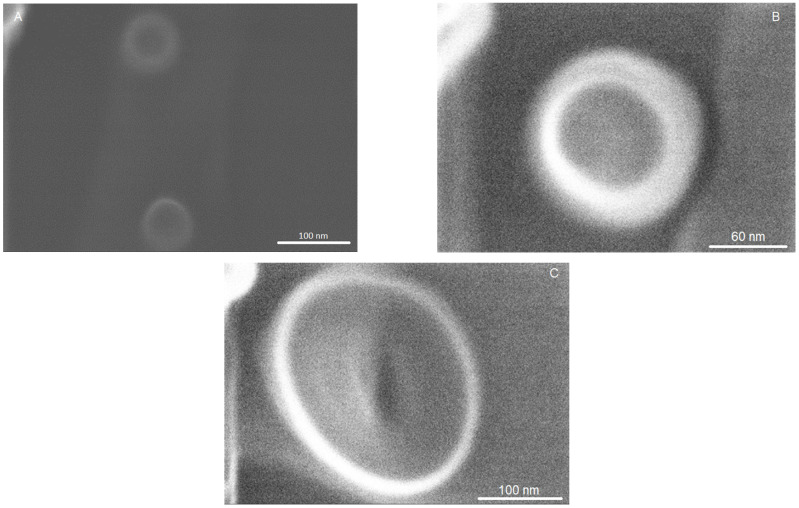

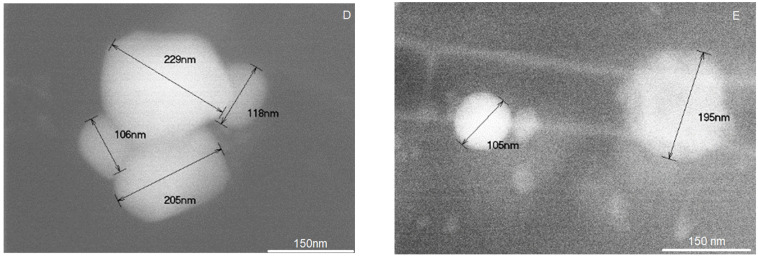
STEM images of NLC−III−Pip−BSA−3 showing the formation of the BSA corona at the surface of the NLC particles(**A**–**C**); STEM images of NLC−III−BSA−0 showing the spherical morphology and the diameter in accordance with DLS analysis (**D**,**E**).

The spherical morphology of NLC-Pip–BSA and NLC-Pip not covered with BSA was viewed in the co-localized STEM micrographs (Figure 6). Using the ZC phase contrast, visible differences between NLC-Pip and NLC-Pip–BSA could be visualized, highlighting the BSA corona surrounding the lipid particle core (Figure 6D,E).

**Figure 7:**
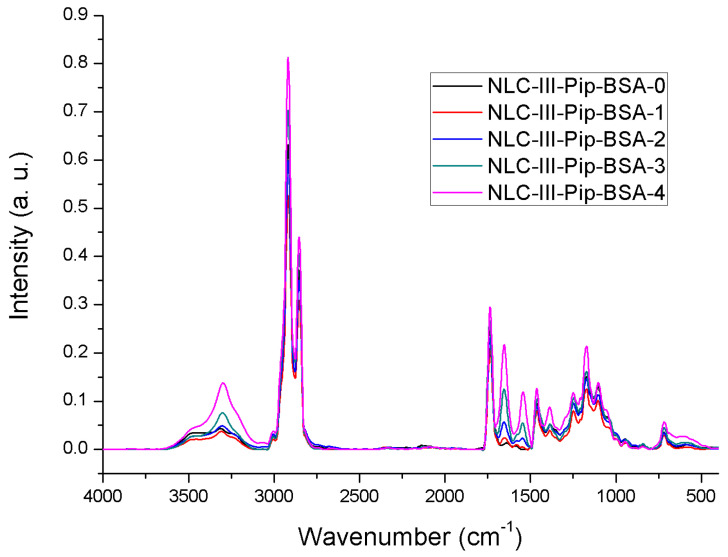
ATR−FTIR analysis of NLCs loaded with Pip and coated with different concentrations of BSA.

**Spectral characterization.** The ATR-FTIR spectra of NLC-Pip–BSA-0/1/2/3/4 were displayed in Figure 7. Generally, all spectra exhibited similar characteristics to the ones of the sample that were not loaded with Pip.

The lowest amount of non-encapsulated Pip, according to the determined high entrapment efficiency percentage, e.g., 81.94% ± 4.17 for NLC-Pip and of 80.45% ± 1.28 for NLC-Pip–BSA, was not detected in the spectrum of the ATR-FTIR analysis. Consequently, by correlating the results of electron microscopy, quantitative assay, and those of the ATR-FTIR analysis, it can be stated that BSA surrounds the solid particles of NLC, while Pip is mostly trapped in the lipophilic lipid core.

**Figure 8:**
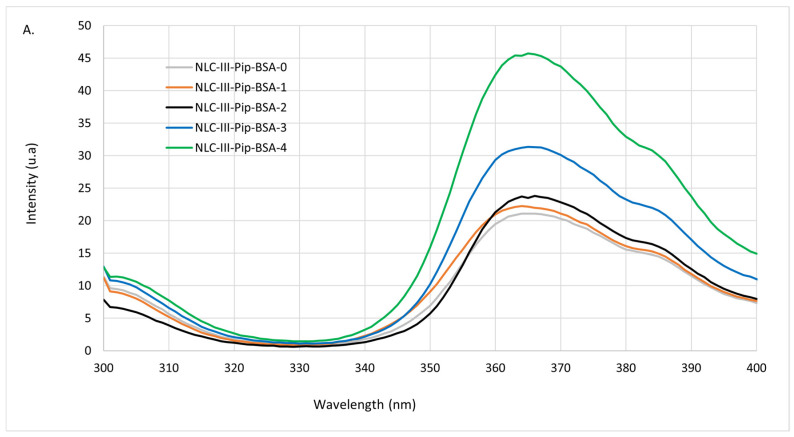

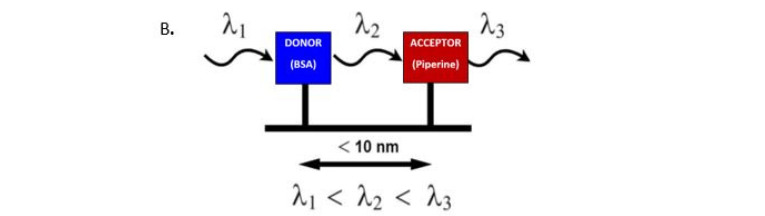
(**A**) Fluorescence emission spectra of NLC-Pip–BSA compared to NLC-Pip; (**B**) the energy transfer process which occurs between the donor (BSA) and acceptor (Pip), when they are very close.

**Fluorescence behavior.** In the fluorescence emission spectra of the hybrid–lipid nanocarriers loaded with Pip, the disappearance of the maximum emission from 325 nm corresponding to BSA was observed (Figure 8A). Fluorescence quenching refers to any process that decreases the fluorescence intensity of a sample such as excited-state reactions, energy transfers, ground-state complex formation and collision processes [42]. These factors can impact the fluorescent activity of BSA. However, simultaneously with the quenching of BSA fluorescence, an interesting fluorescent effect with an emission maximum at 430 nm was detected (Figure 8A). At first view, this emission peak is due to the presence of Pip in the NLC-Pip–BSA; Pip shows a maximum fluorescence emission near 430 nm [43], due to the continuous conjugation between the π electrons of the aromatic ring with the π electrons located in the side chain.

As can be observed from Figure 8A, the emission maximum of NLC-Pip–BSA-1/2/3/4 is influenced by the amount of biopolymer used for coating the lipid nanocarriers. The intensity of NLC-Pip–BSA-1/2/3/4 steadily increases with the increase of BSA concentration. A potential factor responsible for quenching of the intrinsic fluorescence of BSA and the appearance of a relatively intense fluorescence can be correlated with an energy transfer between BSA and Pip. Many-fold enhancement of the fluorescence relative intensity of NLC-Pip–BSA-3 and 4 and the broad blueshift, i.e., 486–464 nm (at high BSA concentration) shows a potential relocation of the fluorophore, because of an energy transfer from BSA to Pip. According to Kaur et al., fluorescence energy transfer is a process by which an excited fluorophore (donor) transfers its energy to an adjacent molecule (acceptor) by dipole–dipole interaction, which is non-radiative [44] (Figure 8B). The molecular mechanism occurring in fluorescence energy transfer could be explained by using the Jablonski diagram:

i. A donor molecule absorbs energy leading to its excitement from the ground state to an excited singlet state. For the excited donor (in our case, BSA), distinct energy states are possible, i.e., spontaneous emission, and non-radiative processes;
ii. If one fluorophore receiver (in our case, Pip) is nearby, the non-radiative energy transfer between the donor and the acceptor can occur. This transfer involves a resonance between the electronic transitions of the two fluorophores, generated by the transition dipole moment for the BSA absorption and a relation of the transition dipole moment to the donor’s emission [44].

This fluorescence energy transfer process occurs when the donor’s emission spectrum could overlap with the acceptor’s absorption spectrum, by means of the distance between the acceptor and donor being short enough (Figure 8B).

### 3.3. The Antitumoral Functionality of NLC-Pip versus Hybrid BSA–Lipid Nanocarriers Loaded with Piperine

Since in a previous comparative evaluation of all hybrid BSA–lipid nanocarriers formulations, NLC-II-BSA-3 showed desired physico-chemical characteristics, e.g., size, physical stability, and suitable fluorescence characteristics, these nanocarriers, NLC-III-Pip without a BSA coating and NLC-III-Pip–BSA-3 were subjected to cell treatments by cytotoxicity, apoptosis, and cell-cycle assays.

A. In vitro cytotoxicity determined for ovarian, breast, and colon tumoral cell lines (MTS and RTCA assays)

**Figure 9:**
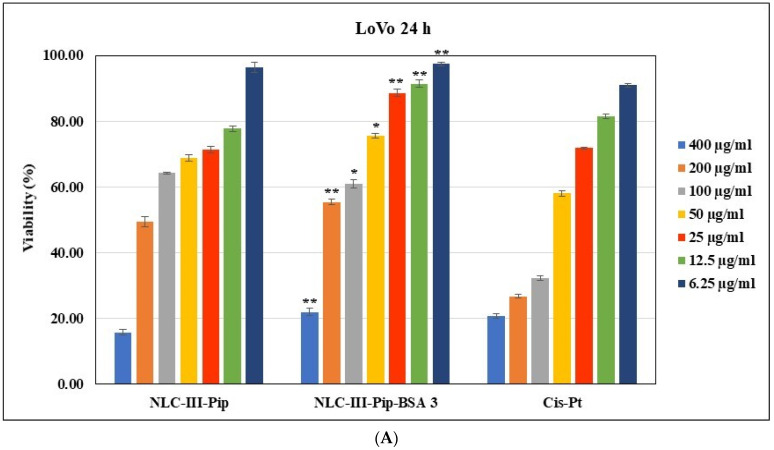

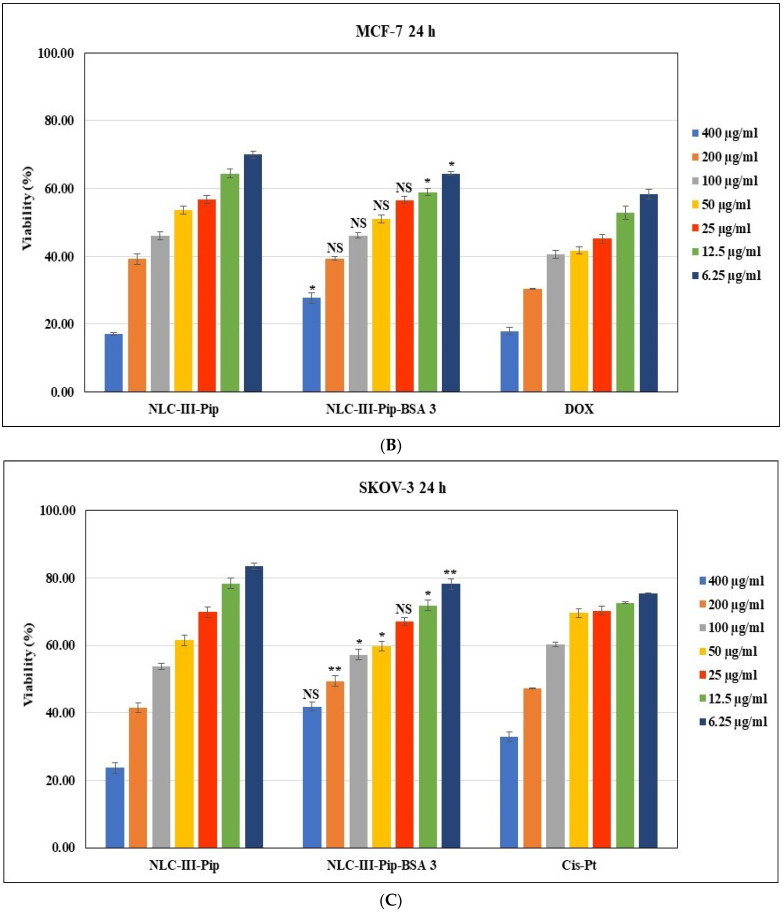

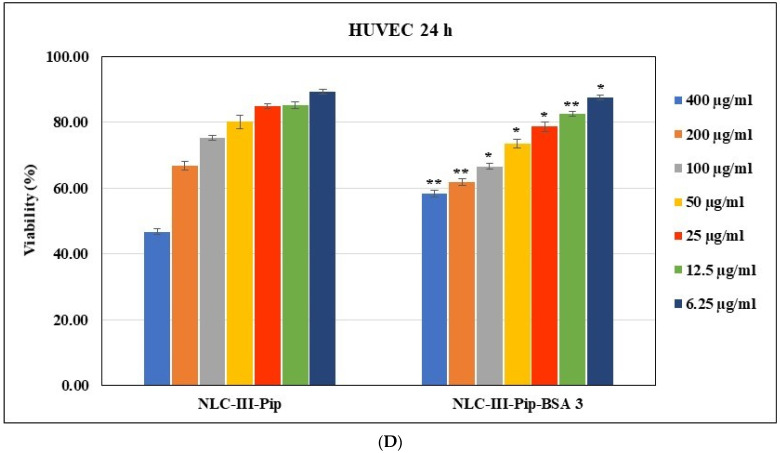
Cell viability of Lovo colon tumor cells (**A**); MCF-7 breast cancer cells (**B**); SKOV-3 ovarian cancer cells (**C**); and normal HUVEC endothelial cells (**D**) after 24 h incubation with NLC-Pip, NLC-Pip–BSA and chemotherapy drugs (Cisplatine/Cis-Pt, Doxorubicin/DOX). (* *p* < 0.05, ** *p* < 0.005). NS = not significant.

Lipid nanocarriers, being composed of physiological and biodegradable lipids, are generally recognized as safe (GRAS), thus assuring an improvement in biocompatibility [45,46]. Starting from Pip antitumoral properties [47], the determination of cell viability against three tumoral cell lines with epithelial-like morphology e.g., LoVo colon tumor cells, MCF-7 breast tumor cells, and SKOV-3 ovarian cancer cell lines, was used to test the cytotoxic effect of NLC formulations. The cytotoxicity of NLC was previously investigated by others in individual cancer cell lines. However, prior to our study, the effects of NLC–BSA in multiple cancer cell lines had not been examined. A preclinical safety study of NLC showed that many cell lines tolerate lipid nanoparticle doses up to 1 mg/mL [48]. Considering the presence of several phytochemicals in the lipid nanocarriers developed in this research—Pip and TO—the evolution of cell viability in normal human umbilical vein endothelial cells (HUVEC) in an in vitro experimental model was also achieved. In an attempt to understand the role of the Pip encapsulation in terms of cytotoxicity, the effect of pure Pip on cell viability after 24h of incubation was studied. The anti-tumoral efficiency of NLC-Pip/NLC-Pip–BSA was comparatively evaluated with two kinds of chemotherapy drugs used to treat several types of cancer—Doxorubicin (DOX) and Cisplatin (Cis-Pt). All formulations showed a concentration-dependent effect, with toxicity increasing proportionally to the NLC-Pip/NLC-Pip–BSA concentration (Figure 9).

For the LoVo colon tumor cells incubated with developed NLC-Pip and NLC-Pip–BSA, a decrease in cell viability was observed, for example, <64% (for concentrations of 100 mg/mL) and <55% (at concentrations of 200 mg/mL). Cytotoxicity induced at these concentrations continued to increase upon treatment of LoVo cell lines with 400 mg/mL. In this last case, it is worth noting that the lipid nanocarriers induced advanced cell death (e.g., 15.73% ± 0.82 cell viability for NLC-Pip and 21.95% ± 1.06 for NLC-Pip–BSA), comparable and even more prominent in the case of NLC-Pip, compared to the cytotoxicity induced by treatment with the chemotherapy drug—Cis-Pt (Figure 9A).

The results obtained for MCF-7 breast tumor cells, the incubation with the two kinds of NLC formulations, with and without BSA, did not significantly affect the MCF-7 cell viability, the same middle cytotoxic effect of around 39% and 46% being determined for both kinds of NLC at concentrations of 100 and 200 mg/mL (Figure 9B). However, a pronounced cytotoxic response was reported for the treatment with 400 mg/mL NLC-Pip of MCF-7 breast cancer-cell lines, e.g., cell viability of 17.18% ± 0.35 for NLC-Pip vs. 18.01% ± 0.93 for Doxorubicin chemotherapeutic drug.

Regarding the ovarian SKOV-3 cancer cells (Figure 9C), cytotoxic concentrations have ranged in the same concentration interval, 100–400 mg/mL, for which cell viability was lower than 65%. Compared to the other two tumor lines, treatment with NLC led to a somewhat higher survival rate, e.g., 23.71% ± 1.59 cell viability for NLC-Pip and 41.81% ± 1.33 for NLC-Pip–BSA. However, the toxicity efficiency induced by NLC was higher in the case of NLC formulations than the toxicity induced by the chemotherapeutic drug, Cisplatin (cell viability of 32.82% ± 1.40 at the same concentration as that in NLC).

In terms of the survival of normal HUVEC cells (Figure 9D), the existence of moderate toxicity was noted at concentrations of 200 and 400 mg/mL, with cell viabilities of 66.7% ± 1.87 for NLC-Pip and 61.91% ± 1.07 for NLC-Pip–BSA, when incubated with a concentration of 200 mg/mL and, respectively, 58.41% ± 0.95 for the treatment with 400 mg/mL NLC-Pip–BSA.

To sum up, under the experimental conditions used, the incubation of BSA-coated–NLC with the selected anti-tumour cell lines affected the viability of the three anti-tumor cell lines in a different manner. For instance, NLC-Pip–BSA showed a more pronounced response against the LoVo colon cell line and MCF-7 breast tumor cell lines than against the ovarian SKOV-3 cell line.

**Figure 10:**
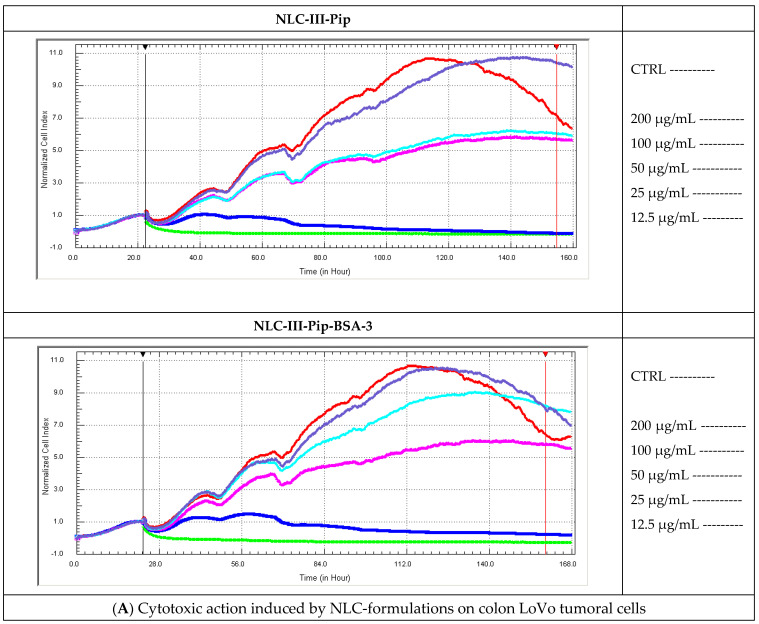

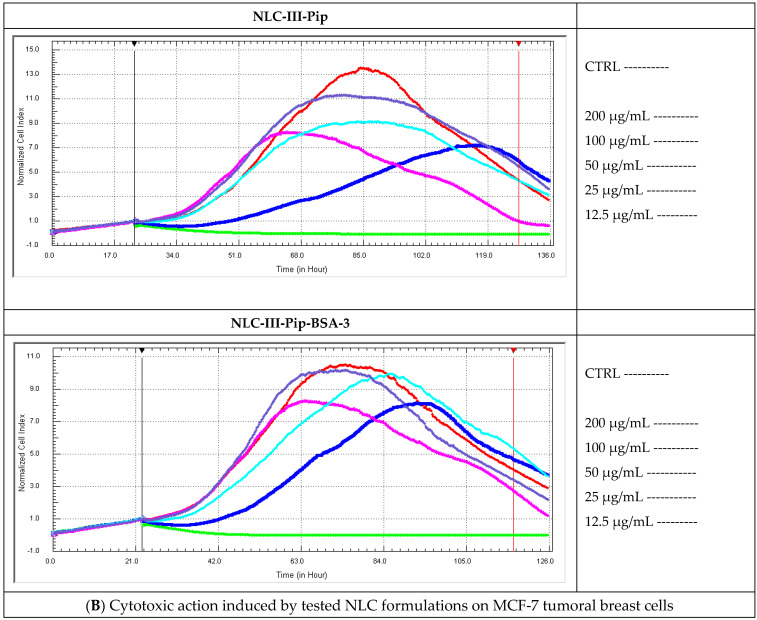

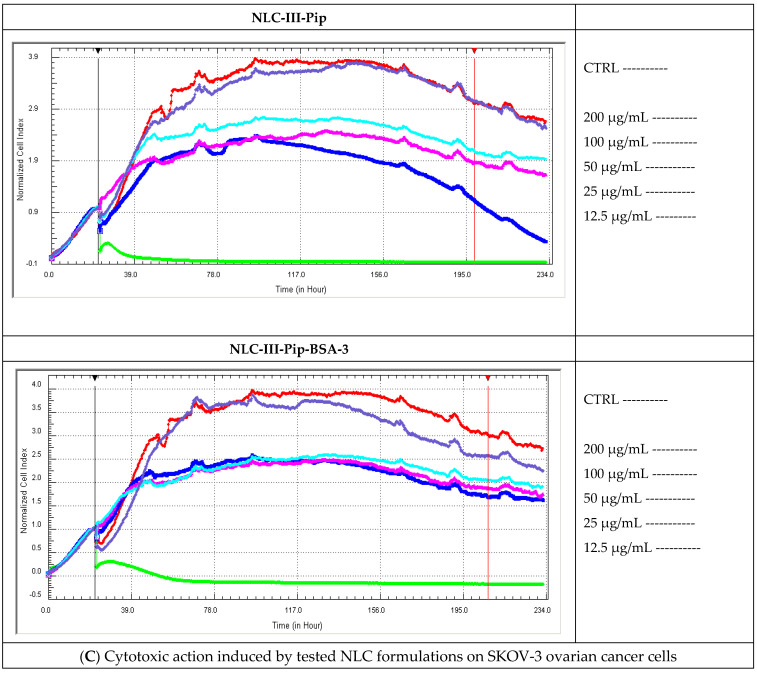
Cytotoxic action vs. proliferative effect induced by NLC−Pip and NLC−Pip−BSA on colon LoVo tumoral cells (**A**); MCF−7 tumoral breast cells (**B**); and SKOV−3 ovarian cancer cells (**C**).

The results of the MTS analysis were completed and confirmed by using the RTCA assay. RTCA has several advantages over MTS colorimetric assay, e.g., minimizing the interference and manifesting the capability of real-time for screening the cytotoxicity of nanoparticles [49]. Thus, the continuous quantitative readout of cells number and monitoring of live cells proliferation in a real-time control for NLC formulations are shown in Figure 10. The results obtained for colon LoVo tumoral cells confirmed the advanced cytotoxicity induced by NLC-Pip and NLC-Pip–BSA, slightly pronounced for NLC-Pip than for BSA-coated NLC-Pip (Figure 10A). This result could be ascribed to a protective effect of the BSA corona shell which could delay the interactions between phytochemical active and cell membranes of LoVo tumoral cells. Concentrations of 100 and 200 mg/mL NLC drastically decreased cell survival (cell death occurred).

In the case of MCF-7 breast tumor cells, the enhanced cytotoxicity occurred only for a concentration of 200 mg/mL. As shown in Figure 10B, the coating of lipid nanocarriers with BSA did not affect the cytotoxic behaviour, a similar trend is detected for NLC-Pip and NLC-Pip–BSA. The incubation of SKOV-3 tumor cells with NLC formulations has revealed a similar moderate cytotoxic action in the range of 25 to 100 mg/mL, while increasing the concentration to 200 mg/mL led to the death of SKOV-3 cells (Figure 10C).

In addition to the MTS and RTCA cytotoxicity study, the concentrations required to inhibit cell proliferation by 50% (IC50) were determined for the corresponding NLC-III-Pip, NLC-III-Pip–BSA-3, Cisplatin and Doxorubicin (Table 2). As can be seen in Table 2, the lipid nanocarriers loaded with Pip had cytotoxic activity against all cancer cell lines, with IC50 values close to those of the cytostatic drugs.

B. The effect of the NLC-Pip and NLC-Pip–BSA on the apoptosis process of colon, breast and ovarian tumor cells

**Figure 11:**
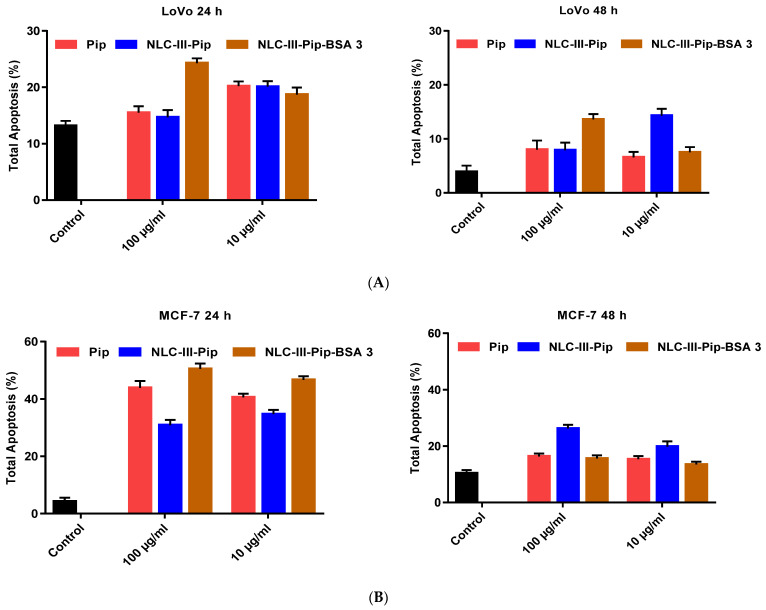

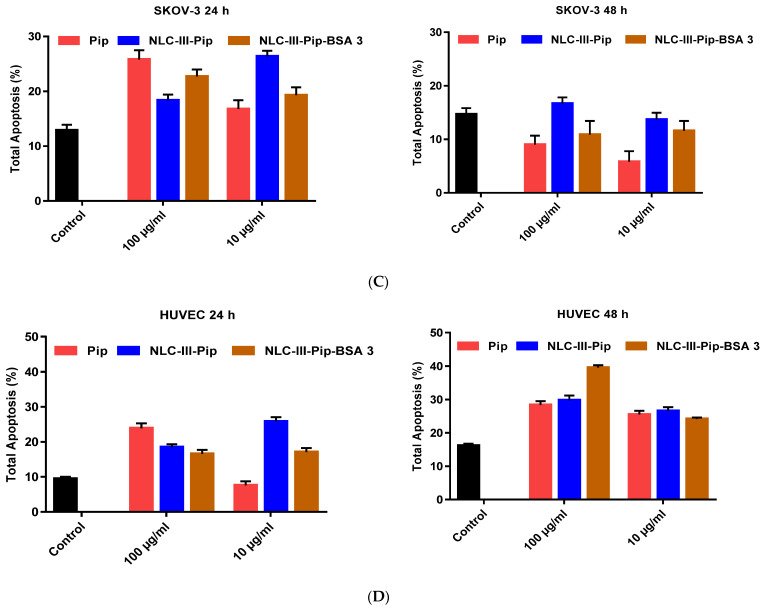
Apoptosis of normal HUVEC cells (**A**); LoVo colon cancer cells (**B**); MCF-7 breast cancer cells (**C**); and SKOV-3 ovarian cancer cells (**D**) induced by 24 and 48 h treatment with the NLC-III-Pip and NLC-III-Pip–BSA-3.

The culture of untreated LoVo colon tumor cells shows that they do not enter the apoptotic process, which means the apoptotic process is almost totally inhibited. Treatment of LoVo tumor cells for 24 h with NLC-Pip (10 μg/mL) and NLC-Pip–BSA (100 μg/mL) increase the apoptosis by 1.5× and 2× (Figure 11A). An extended treatment period (48 h) of LoVo cells with the NLC-Pip and hybrid NLC-Pip–BSA, did not lead to noticeable changes. A comparative view on apoptosis modulation showed that NLC-Pip–BSA (100 μg/mL) was more effective than NLC-Pip, the first hybrid–lipid nanocarriers leading to 25% (24 h) apoptosis (Figure 11A).

The application of NLC-Pip on MCF-7 breast tumor cells caused a significant increase in apoptosis of ~8×, while NLC-Pip–BSA showed an 11-fold increase in apoptosis compared to untreated cells (Figure 11B). For instance, NLC-Pip–BSA (100 μg/mL) led to 50% apoptosis in breast MCF-7 cells after 24 h treatment. Since the differences between the level of apoptosis induced by NLC-Pip–BSA (100 μg/mL) and NLC-Pip–BSA (10 μg/mL) did not differ significantly, it could be concluded that NLC-Pip–BSA is effective in activating the apoptotic process of MCF-7 tumor cells compared to the control.

The significant tumor inhibition encountered for both, MCF-7 and LoVo cells, can be attributed to BSA, which binds to different protein receptors overexpressed in the membrane of breast and colon tumor cells, which would lead to an improvement in the cellular internalization of NLC-Pip–BSA in the tumor sites. Previous cellular uptake studies have shown that BSA binds preferentially to the tumoral glycoproteins-60 and SPARK receptors and increases the specific absorption of albumin-based nanoparticles in cancer cells [12]. This albumin–tumoral protein receptor interaction facilitated the internalization of albumin-NPs by endocytosis (caveolae-mediated pathway) or via micropinocytosis [50]. It can be presumed that there is an improved cellular uptake of NLC-Pip–BSA by MCF-7 cells (through endocytosis or direct penetration), which may increase the intracellular accumulation of Pip. In other related studies, it has been demonstrated that natural compounds-induced apoptotic tumoral cell death could be related to an increase in death receptors expression [51]. For instance, various phytochemicals, e.g., curcumin, resveratrol, epigallocatechin-3-galat, and capsaicin inhibited ovarian, liver, prostate, and leukemic cancer cell growth through p53-dependent induction of apoptotic cell death [52,53].

The treatment of SKOV3 ovarian tumor cells for 24 h with NLC-Pip (10 μg/mL) induced a 2× higher apoptosis increase compared to the control and acts in the same way with NLC-Pip–BSA (100 μg/mL) (Figure 11C). Extending the treatment period to 48 h led to the observation that the Pip-loaded nanocarriers no longer influenced the apoptotic process of SKOV3 cells. Compared to the first tumor lines, MCF-7 and LoVo, in the case of SKOV3 tumor cells, a more potent action of NLC-Pip than NLC-Pip–BSA was detected in inducing apoptosis. This slightly different behavior of NLC-Pip versus NLC-Pip–BSA on SKOV3 cells could be a consequence of the different charges of the two types of nanocarriers. The coating of nanocarriers with BSA consumes the cationic charges of phosphatidylcholine and can lead to difficulty in the internalization of NLC-Pip–BSA in tumor cells. Similarly, Zhao et al., demonstrated that targeting of SKOV3 was improved with cationic cholesterol-based lipid nanoparticles for the gene therapy of ovarian cancer [54].

Regarding the normal HUVEC cells, the treatment with NLC-Pip or NLC-Pip–BSA has revealed the presence of a moderate apoptosis process (Figure 11D). The treatment with 100 μg/mL NLC-Pip or NLC-Pip–BSA causes an increase in apoptosis of ~ 2× at 24 h. After 48 h of treatment, 100 μg/mL NLC-Pip induced a 1.8× increase in apoptosis compared to the control, while NLC-Pip–BSA (100 μg/mL) seemed to affect the apoptotic process more, inducing a 2.5× increase compared to the untreated cells.

C. The effect of the Piperine-loaded nanocarriers on the cell cycle of normal and tumor cells.

**Figure 12:**
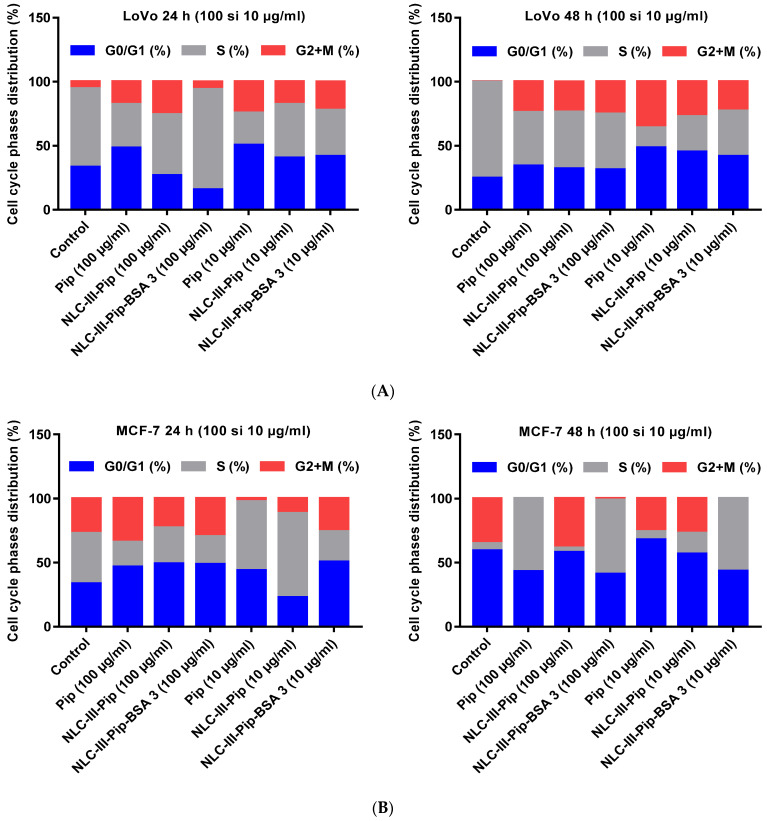

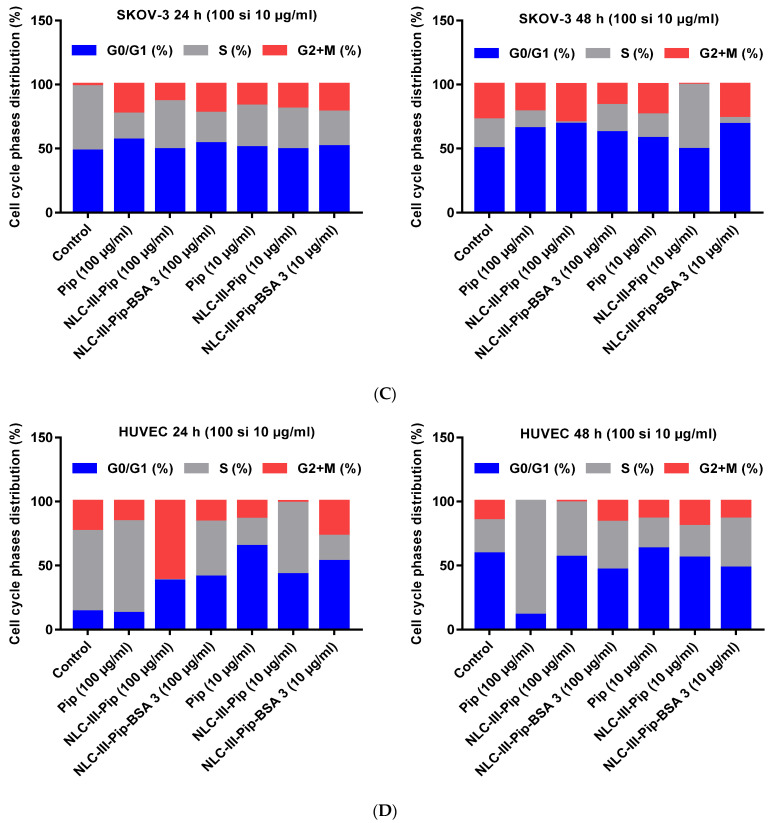
Cell cycle modulation of various tumoral cells: (**A**) Lovo colon tumor cells; (**B**) MCF-7 breast tumor cells; (**C**) SKOV-3 ovarian tumor cells; and (**D**) HUVEC normal cells after 24 h and 48 h of treatment with NLC-III-Pip and NLC-III-Pip-BSA-3.

The effect induced by the lipid and hybrid nanocarriers on the cell cycle of different tumor cells was comparatively analyzed by flow cytometry. The results obtained for LoVo colon tumor cells treated with NLC-Pip, indicated that both concentrations significantly decreased the S phase of the cells cycle, accompanied by an increase in the G2 + M phase (Figure 12A). It is worth noting that covering NLC with albumin does not always lead to a significant disruption of the tumor cell cycle. For these colon tumoral cells, the most efficient treatment seemed to be NLC-Pip–BSA (at 10 μg/mL, 24 h), while after 48 h the maximum inhibition effect was transferred to NLC-Pip (Figure 12A).

An interesting event of the cell-cycle behavior was noticed after 48h of treatment: the treatment with 100 μg/mL NLC-Pip and NLC-Pip–BSA ensured a similar distribution of cell-cycle phases. The way in which the NLC-Pip–BSA (100 μg/mL) acted on the development of the cell cycle showed that it has a different influence on the S phase of the cell cycle, by means of a significant decrease of the S proliferative phase, e.g., S = 43.6%, as compared to the control, e.g., S = 75.07%.

NLC-Pip–BSA (10 and 100 μg/mL) affected the cell-cycle phase of MCF-7 breast tumor cells after 24 h of treatment. Application of NLC-Pip–BSA resulted in a decrease in the S proliferative phase (e.g., 23.76% for a concentration of 10 μg/mL and 21.8%, for a concentration of 100 μg/mL, respectively), as compared to the control (S = 39.45%). Although after 24 h, treatment with NLC-Pip did not significantly influence the MCF-7 tumor cycle, after 48 h, NLC-Pip (100 μg/mL) becomes more efficient in decreasing the proliferative S phase (Figure 12B).

Treatment of SKOV3 ovarian tumor cells with conventional– and hybrid–NLC-Pip showed a noticeable tumor suppressor effect, underlined by a decrease of the S phase. NLC-Pip (100 μg/mL) and NLC-Pip–BSA (100 μg/mL) decreased the S phase of the cell cycle after 24 h treatment, but at 48h, the maximum inhibition effect of the S phase (S = 1.13%) was exerted by NLC-Pip (100 μg/mL) versus the control (S = 22.4%). This effect is also supported by previous results on the apoptosis process (included in Section 3.3*.B*), which underlines the high potential of the two categories of NLC-Pip as tumor suppressors and in decreasing tumor progression.

The cell-cycle analysis of HUVEC normal cells showed that after 24 h, the cells are in the maximum proliferation phase supported by the S phase value of 62.74% for the untreated cells (Figure 12D). Treatment with 10 μg/mL NLC-Pip or NLC-Pip–BSA changed the distribution of the cell-cycle phases by recording a decrease in the S phase and a balanced distribution of G1/G2 and G2+M phases. After 48 h of treatment, hybrid–NLC significantly affects the cell-cycle phases. For instance, an evident increase in the S% proliferation phase was induced by NLC-Pip–BSA in normal HUVEC cells (at both concentrations, 10 and 100 μg/mL as compared to that of the control cells (Figure 12D). The lower proliferation phase of normal HUVEC cells occurred in the case of NLC-Pip (100 μg/mL) doubled by an incentive cell proliferation detected for the treatment with 100 mg/mL NLC-Pip–BSA, underlining the improved proliferation effect of nanocarriers coated with BSA.

## 4. Conclusions

We designed a new delivery platform consisting of BSA–lipid nanocarriers loaded with Pip (NLC-Pip–BSA) for improved cellular internalization in different tumor cells. These lipid nanostructured systems presented an adequate surface charge for the creation of non-covalent interactions between the shell formed by the ionic surfactants (PC and AOT) and the BSA. It was observed that the ratio of surfactants influenced the binding of BSA to the surface of the lipid nanocarriers. Along with the creation of electrostatic interactions and hydrogen bonds, there was a noticeable change in the native structure of albumin (highlighted by spectral characterization in ATR-FTIR and fluorescence spectroscopy). The distribution of the Pip in the lipid core was confirmed by ATR-FTIR spectroscopy and fluorescence spectroscopy. Many-fold enhancement of the fluorescence relative intensity of NLC-Pip–BSA showed a potential relocation of the fluorophore because of an energy transfer from BSA to Pip. DLS and electron microscopy confirmed that BSA-coated NLC-Pip had a size of 50 ÷ 200 nm, a near-spherical morphology and a strongly electronegatively charge of −50 mV.

According to MTS and RTCA, the lipid nanocarriers induced advanced LoVo colon tumor cell death (e.g., 15.73% ± 0.82 cell viability and 21.95% ± 1.06 for 400 mg/mL NLC-Pip and NLC-Pip–BSA, respectively), comparable and even more prominent than those produced by the chemotherapy drug, Cisplatin. The application of NLC-Pip on MCF-7 breast tumor cells caused a significant increase in apoptosis of ~8×, while NLC-Pip–BSA was shown to have an 11-fold increase in apoptosis compared to untreated cells. A flow cytometry assay revealed that 100 μg/mL NLC-Pip–BSA (48 h) differentially influenced the proliferative S phase of the LoVo cell cycle, recording a significant decrease in the proliferative S phase, e.g., S = 43.6%, compared to the control, e.g., S = 75.07%. Treatment of SKOV3 ovarian tumor cells with untargeted- and targeted BSA-coated–NLC-Pip also showed a noticeable tumor-suppressor effect. NLC-Pip (100 μg/mL) and NLC-Pip–BSA (100 μg/mL) decreased the S phase of the cell cycle after 24 h treatment, but at 48 h, the maximum inhibition effect of the S phase (S = 1.13%) was exerted by NLC-Pip (100 μg/mL) versus the control (S = 22.4%). Our results demonstrated that in response to NLC, various cell types differently utilize distinct apoptotic pathways. One of the likely mechanisms responsible for promoting apoptosis and cell-cycle modulation could be attributed to albumin, which binds to glycoprotein receptors overexpressed in the membrane of MCF-7 breast and LoVo colon tumor cells and leads to an improvement in the cellular internalization of NLC-Pip–BSA in the tumor sites. Due to the innovativeness of the research, it is difficult to interpret the results and determine their clinical significance, but nevertheless, the in vitro results imply that BSA-based lipid nanocarriers may be a good vector for Pip delivery in vivo.

## Figures and Tables

**Table 1:** Composition of NLCs (**A**) and BSA-coated NLCs formulations (**B**).

(A)							
NLCs Formulations	Lipid Phase				Aqueous Phase		
	GSM (g)	CB (g)	TO (g)	Pip (g)	AOT (g)	PC (g)	TW20 (g)
NLC-I	3.5	3.5	3	-	0	0.3	1.7
NLC-II	3.5	3.5	3	-	0.3	0.7	1
NLC-III	3.5	3.5	3	1	0.3	0.7	1
**(B)**							
**BSA-Coated** **NLCs Formulations**	**BSA Solutions** **Conc. (mg/mL)**				**BSA: NLC Ratio** **(% wt.)**		
NLC-I/II/III-BSA-1	1				0.01		
NLC-I/II/III-BSA-2	5				0.05		
NLC-I/II/III-BSA-3	10				0.1		
NLC-I/II/III-BSA-4	20				0.2		

**Table 2:** IC50 values (μg/mL) for NLC-III-Pip, NLC-III-Pip-BSA-3, Cisplatine and Doxorubicin.

NLC Formulations and Drugs/Cell Lines	IC50 (μg/mL)			
	HUVEC	LoVo	MCF-7	SKOV-3
NLC-III-Pip	231.10 ± 2.1	191.76 ± 1.3	109.42 ± 1.6	177.00 ± 1.1
NLC-III-Pip-BSA-3	292.43 ± 1.9	224.67 ± 1.1	209.13 ± 1.4	247.88 ± 1.2
Cis-Pt	-	144.25 ± 1.2	-	113.45 ± 0.9
DOX	-	-	12.11 ± 1.0	-

## Data Availability

Not applicable.

## Supplementary Materials

The following supporting information can be downloaded at: https://www.mdpi.com/article/10.3390/pharmaceutics15041125/s1.
