# Supplementary material for: Novel Bovine Serum Albumin-Decorated–Nanostructured Lipid Carriers Able to Modulate Apoptosis and Cell-Cycle Response in Ovarian, Breast, and Colon Tumoral Cells
Source: Pharmaceutics. 2023 Apr 2;15(4):1125. doi: 10.3390/pharmaceutics15041125 (PMC10144507; doi:10.3390/pharmaceutics15041125)
Supplement: Supplementary file 1 [file pharmaceutics-15-01125-s001.zip › pharmaceutics-2248887-supplementary.pdf]

pharmaceutics

Supplementary Material: Novel Bovine Serum Albumin-Decorated–Nanostructured Lipid Carriers Able to Modulate Apoptosis and Cell-Cycle Response in Ovarian, Breast, and Colon Tumoral Cells

Robert Tincu, Mirela Mihaila, Marinela Bostan, Florina Teodorescu, Daniela Istrati, Nicoleta Badea and Ioana Lacatusu

S1. Materials and methods

S1.1. Cell cultures conditions and treatments

The cancer cell lines used in this study are LoVo (human colon adenocarcinoma), MCF-7 (human breast adenocarcinoma), and SKOV-3 (human ovarian adenocarcinoma), which were purchased from the American Type Culture Collection (ATCC). The normal cell line used as a reference is human umbilical vein endothelial cells (HUVEC). The cells were maintained in culture using DMEM: F12 medium supplemented with 2mM L Glutamine, 10% fetal bovine serum, 100 units/ml penicillin, 100 μg/ml streptomycin (Sigma Aldrich, St. Louis, Mo, USA) and were incubated at 37°C in a 5% CO2 humidified atmosphere. When the cells reached around 60% confluence, they were treated with different concentrations of compounds for various periods of time. After treatment, the cells were detached using a non-enzymatic solution of PBS/1 mM EDTA, washed twice in PBS, and used for proliferation/cytotoxicity assays or evaluation of apoptotic events by flow cytometry. Alternatively, cells were fixed in ice-cold ethanol/PBS (70:30) and kept at 4°C until used for cell cycle analysis by flow cytometry. The control cells were untreated cells used as a reference for comparison in all experiments described in this study.

S1.2. Characterization methods

S1.2.1. Particle size and polydispersity index

Dynamic light scattering (DLS) was used to determine the mean particle size (Zave) and polydispersity index (PdI) of the conventional NLC and of BSA-coated NLC using a Zetasizer Nano ZS (Malvern Instruments Ltd., Worcestershire, UK) at a scattering angle of 90° and a temperature of 25°C. To acquire a sufficient scattering intensity, samples were obtained by diluting the lipid nanocarrier dispersions with water. The particle size data were evaluated using intensity distribution. Each value of Z_{ave} and PdI was given as the average of three individual measurements.

S1.2.2. Zeta-potential measurements

The electrical characteristics of the NLC-Pip and NLC-Pip-BSA were determined using the electrophoretic light scattering method (Zetasizer Nano ZS, Malvern Instruments Inc., Worcestershire, UK). The zeta potential, ξ, was determined (in a capillary cell) by using the Helmholtz–Smoluchowski equation ($\mu e = \xi\varepsilon/\eta$ with, ε and η being the dielectric constant and viscosity of the solvent, respectively). The measured particle electrophoretic mobility (μe) was converted into zeta potential (ξ). Prior to analysis, the NLC dispersions (100 μL) were diluted with water (25 mL), and the conductivity was corrected to 50 μS/cm with a solution of 0.9% NaCl. A triplicate of each measurement was made.

S1.2.3. Morphological characteristics

The morphological characteristics of NLC-Pip-BSA were observed using Transmission Electron Microscopy (TEM). A Hitachi HD 2700 Scanning Transmission Electron Microscope (Hitachi High-Tech Corporation, Tokyo, Japan) was utilized to conduct the TEM study. For the proper TEM examination, aqueous NLC-Pip and NLC-Pip-BSA dispersions were diluted in distillate water and placed on standard Cu grids with Carbon thin layer coating. Bright field mode was used to get the TEM data.

S1.2.4. Spectroscopic characterization (FTIR)

Fourier Transform Infrared (FTIR) spectra of the powdered NLC were recorded on a Bruker Vertex 70 Spectrometer, with a horizontal device for attenuated reflectance and diamond crystal, on a spectral window ranging from 4000 to 400 cm^{-1}.

S1.2.5. Fluorescence assay

Fluorescence Spectra of the NLC-Pip and NLC-Pip-BSA were recorded on FP-650 Spectrofluorometer Jasco (Tokyo, Japan) equipped with a microcomputer for data recording. The samples were illuminated with a 285 nm excitation light and the fluorescence spectra were recorded.

S1.2.6. Entrapment efficiency

The entrapment efficiency of Pip in NLC formulations was determined using the UV-Vis spectroscopy method. An amount of 0.15 g of lyophilized NLC_III formulation was gently mixed with 1 mL of ethanol and centrifuged at 15000 rpm for 15 minutes [31, 32]. The supernatant containing the unentrapped piperine was diluted to 25 mL and analyzed using a UV-Vis Spectrophotometer V670 Jasco (Tokyo, Japan) at λ = 342 nm. The percentage of entrapment efficiency was calculated using the equation:

$$EE\% = \frac{W_i - W_a}{W_i} \cdot 100 \qquad (1)$$

where Wi – the initial amount of piperine and Wa – the analyzed amount of piperine in the supernatant.

S1.2.7. Real-Time Cell Analysis of NLC-Pip and NLC-Pip-BSA – Treated Cells

The real-time cell analysis (RTCA) assay and xCELLigence DP-System were used to monitor cell growth continuously. The xCELLigence DP-System is a label-free, cell-based in vitro assay that allows real-time monitoring of cellular processes such as cell viability and cytotoxicity [33]. The cell index (CI), which is a quantitative measure of the cell number present in a well, changes when there is a change in a cell's status, such as cell morphology, cell adhesion, or cell viability. Cells were cultured in DMEM culture medium supplemented with 2 mM L-glutamine and 10% FCS and seeded in 100 µL culture medium in 16 E-Plates cells (ACEA Biosciences, USA). Growth curves were automatically recorded in real-time on the xCELLigence System with a DP device after cells proliferated until a cellular index (CI) over 1.0, usually after 24 h. After this point, the compounds studied were added, and growth curves were registered in real-time on a computer using RTCA 2.1.2. Software. By monitoring the changes in the CI of cells over time, it is possible to generate compound-specific profiles that are dependent on the

biological mechanisms of action of each compound. This approach enables us to evaluate the effects of compounds on cell proliferation and to identify potential cytotoxic effects.

S1.2.8. Assessment of Cytotoxicity by MTS Assay

The assay used in this experiment is a CellTiter 96 Aqueous One Solution Cell Proliferation Assay (Promega, Madison, WI, USA), which is a type of MTS colorimetric assay [34]. This assay measures the ability of metabolically active cells to reduce MTS, a yellow tetrazolium salt, to a colored formazan that is soluble in the culture medium. The experiment was conducted in triplicate using 96-well microtiter plates with flat-bottom (Falcon). 1.5 × 104 cells/well were cultured in 100 μL for 24 h, after which culture supernatants were discarded. Then, the cells were treated with increasing concentrations of the compounds being studied for an additional 24 h and 48 h. After the incubation period, a reagent mixture of MTS [3-(4,5-dimethylthiazol-2-yl)-5-(3-carboxymethoxy-phenyl)-2-(4-sulfophenyl)-2H-tetrazolium, inner salt] and PES (phenazine ethosulfate) was added to each well, and the plates were incubated for 4 hours at 37°C with mild agitation every 20 minutes. The color that developed during the incubation period was measured spectrophotometrically at λ = 492 nm using a Dynex ELISA reader (DYNEX Technologies-MRS, USA). To calculate the percentage of viability compared to untreated cells (considered 100% viable), the following formula can be used:

$$\text{Viability (\%)} = [(T - B) / (U - B)] \times 100$$

Where: T = absorbance of treated cells, U = absorbance of untreated cells and B = absorbance of culture medium (blank), for λ = 492 nm

An experiment involving cell lysis was performed and results were measured as means with standard deviations. In addition, a parallel test was performed to evaluate the cytotoxicity of DMSO (dimethyl sulfoxide) under the same experimental conditions. No cellular cytotoxicity was observed for DMSO concentrations lower than 1%, which was not shown in the data provided.

S1.2.9. Apoptosis assay by Flow Cytometry

The methodology to carry out an apoptosis assay uses the Annexin V-FITC Apoptosis Detection Kit from Becton Dickinson (BD) Biosciences [35, 36]. First, 1 × 10^5 cells per tube were resuspended in 100 μL binding buffer. Then, the cells were stained simultaneously with 5 μL FITC Annexin V (green fluorescence) and 5 μL propidium iodide (PI) in the dark, at room temperature for 15 min. After the staining, 400 μL binding buffer was added to each tube. The percentages of apoptotic events were measured by using data acquisition by flow cytometry using a FACS Canto II cytometer (Becton Dickinson, Immunocytometry System, Mountain View, CA, USA). The analysis was done using DIVA 6.2 software in order to discriminate viable cells (FITC−PI−) from necrotic cells (FITC+PI+) and early apoptosis (FITC+PI−) from late apoptosis.

S1.2.10. Cell Cycle Analysis by Flow Cytometry

The protocol for analyzing the cell cycle phase distribution of ethanol fixed cells (1 × 10^6) using flow cytometry, involves the following steps: the fixed cells are washed twice in PBS; cell pellets are resuspended in 350 μL of PBS and add 50 μL of 10 mg/mL RNase A (1 mg/mL final concentration); incubate the cells at 37°C for 10 minutes; it adds 100 μL of 100 mg/mL PI solution (20 μg/mL final concentration) and incubate the cells at 37°C for 10 minutes. Until data acquisition, the probes were kept in the dark at 4°C. Is collected a minimum of 20000 events for each sample using a FACS Canto II flow cytometer (BD Immunocytometry System, Mountain View, CA). To analyze the data and determine the cell cycle phase distribution after debris exclusion, is used ModFIT software (Becton Dickinson).

Reference

31. Lacatusu, I.; Iordache, T.A.; Mihaila, M.; Mihaiescu, D.E.; Pop, A.L.; Badea, N. Multifaced role of dual herbal principles loaded-lipid nanocarriers in providing high therapeutic efficacity. *Pharmaceutics* **2021**, *13*, 1511. https://doi.org/10.3390/pharmaceutics13091511.

32. Lacatusu, I.; Badea, N.; Udeanu, D.; Coc, L.; Pop, A.; Cioates Negut, C.; Tanase, C.; Stan, R.; Meghea, A. Improved anti-obesity effect of herbal active and endogenous lipids co-loaded lipid nanocarriers: Preparation, in vitro and in vivo evaluation. *Mat. Sci. Eng. C* **2019**, *99*, 12–24. https://doi.org/10.1016/j.msec.2019.01.071.

33. Stecoza, C.E.; Nitulescu, G.M.; Draghici, C.; Caproiu, M.T.; Olaru, O.T.; Bostan, M.; Mihaila, M. Synthesis and anticancer evaluation of new 1,3,4-oxadiazole derivatives. *Pharmaceuticals* **2021**, *14*, 438–453. https://doi.org/10.3390/ph14050438.

34. Mihaila, M.; Hotnog, C.M.; Bostan, M.; Munteanu, A.C.; Vacaroiu, I.A.; Brasoveanu, L.I.; Uivarosi, V.; Anticancer activity of some ruthenium (III) complexes with quinolone antibiotics: In vitro cytotoxicity, cell cycle modulation, and apoptosis-inducing properties in LoVo colon cancer cell line. *Appl. Sci.* **2021**, *11*, 8594–8613**.** https://doi.org/10.3390/app11188594.

35. Botezatu, A.; Iancu, I.V.; Plesa, A.; Manda, D.; Popa, O.; Bostan, M.; Mihaila, M.; Albulescu, A.; Fudulu, A.; Vladoiu, S.V.; et al. Methylation of tumour suppressor genes associated with thyroid cancer. *Cancer Biomark.* **2019**, *25*, 53–65. https://doi.org/10.3233/CBM-182265.

36. Bostan, M.; Petrica-Matei, G.G.; Ion, G.; Radu, N.; Mihaila, M.; Hainarosie, R.; Brasoveanu, L.I.; Roman, V.; Constantin, C.; Neagu, M.T. Cisplatin effect on head and neck squamous cell carcinoma cells is modulated by ERK1/2 protein kinases. *Exp. Ther. Med.* **2019**, *18*, 5041–5051. https://doi.org/10.3892/etm.2019.8139.
